# External Load Variability in Elite Futsal: Positional Demands and Microcycle Structuring for Player Well-Being and Performance

**DOI:** 10.3390/sports13010007

**Published:** 2025-01-02

**Authors:** Héctor Gadea-Uribarri, Elena Mainer-Pardos, Ainhoa Bores-Arce, Rafael Albalad-Aiguabella, Sergio López-García, Carlos Lago-Fuentes

**Affiliations:** 1 Faculty of Education, Universidad Pontificia de Salamanca, 37002 Salamanca, Spain; hgadeauribarri@hotmail.com (H.G.-U.); slopezga@upsa.es (S.L.-G.); 2 Health Sciences Faculty, Universidad San Jorge, Autov A23 km 299, Villanueva de Gállego, 50830 Zaragoza, Spain; ralbalad@usj.es; 3 Faculty of Health Sciences, Universidad Europea del Atlántico, 39011 Santander, Spain; ainhoa.bores@uneatlantico.es (A.B.-A.); carlos.lago@uneatlantico.es (C.L.-F.)

**Keywords:** load monitoring, periodisation, team sport, player position

## Abstract

The aim of this study was to compare the external load of each session along competitive microcycles on an elite futsal team, considering the positions and relationships of the players. The external load of 10 elite players from a First Division team in the Spanish Futsal League (age 27.5 ± 7 years, height 1.73 ± 0.05 m, weight 70.1 ± 3.8 kg) were recorded across 30 microcycles. The players’ external loads were monitored using OLIVER devices. To analyse the external load, Levene’s test was conducted to assess the homogeneity of variances, followed by one-way analysis of variance (ANOVA) to identify differences in dependent variables across the different microcycle days and player positions. Regarding external load during the microcycle, the day with the lowest external load was MD-1, and the days with the highest external load were MD-3 and MD-4. In addition, considering playing positions, pivots exhibited the lowest loads throughout the microcycle, whereas wingers and defenders exhibited the highest loads, depending on the variables analysed. By providing reference values from elite contexts, this study offers practical insights for S&C coaches to optimize microcycles. Furthermore, it contributes to workload management strategies within sport science and public health frameworks, promoting sustainable performance and athlete wellness in futsal.

## 1. Introduction

Recent advances in data collection and analysis technologies have enhanced our understanding of the workload and physical demands of team sports [1]. These developments have enabled teams to refine their training programmes, thereby improving player performance and reducing injury risks in elite athletes [2,3]. Monitoring workload has become a crucial aspect of sports research, providing a solid foundation for informed decision-making and injury prevention [4,5]. Over more than two decades of study and application in various sports, the concept of training load has evolved to include both external loads (physical exertion performed during a training session or match) and internal loads (related to physical and physiological responses) [6,7].

From a multidisciplinary perspective, understanding the intricate relationships between workload, performance, and injury prevention in sport aligns with broader public health goals, such as promoting athlete wellness and longevity in sport careers. This highlights the importance of integrating sport science and public health frameworks to ensure sustainable sport development.

The global rise in futsal popularity over the past 15 years has been remarkable [8]. Despite this growth, published studies on futsal are limited [1,9,10,11]. For this reason, it is necessary to continue investigating the training methods employed by teams, monitoring load strategies and coaching staff strategies to better understand the physical demands of this intermittent sport [8]. In futsal, external workload monitoring is essential for tracking both training sessions and matches. Data have shown that male players cover an average distance of 4000 m per match, equivalent to approximately 120 m per minute [12], 675 m at high intensity (12–18 km/h) [1], 135 m at maximum intensity (>18 km/h) [10], 534 m in accelerations (>2 m/s^2^), and 510 m in decelerations (>2 m/s^2^) [13].

Training sessions across microcycles were designed to adapt player performances throughout the season. They can improve the physical attributes of players by aligning their capabilities with the sport’s specific demands [8]. However, excessive training without sufficient recovery time can be detrimental, and insufficient workload can negatively impact and limit both individual and collective performance capabilities [7]. Therefore, developing strategies to monitor and control the workload during various training sessions is crucial for enhancing performance and reducing the risk of injury [8].

Furthermore, players must be well-prepared to meet the demands of the sport. Adapting training plans to adjust volume and intensity throughout competitive cycles is essential [14]. New technologies, such as global positioning systems (GPS) and inertial measurement units (IMU), have provided coaches with tools to precisely monitor the external workload experienced by players. These technologies allow for the tailoring of training sessions based on the positions of players and individual needs [10,15].

The use of advanced monitoring technologies not only improves player-specific training, but also addresses critical public health issues by reducing the prevalence of injury and promoting optimal recovery. These innovations bridge the gap between research and practice, exemplifying the multidisciplinary spirit of contemporary sports science.

Another key factor in improving performance is maintaining an appropriate balance between training and recovery. However, planning training programmes for team sports presents challenges, such as determining the appropriate workload distribution during weekly training sessions and during competitive phases [15]. To achieve a more personalised approach, considering workload data specific to players’ positions would be beneficial, although such data are currently limited in futsal [14].

Knowledge of workload variations not only contributes to optimizing sport performance, but also aligns with public health priorities, ensuring the long-term health of athletes and sustainable participation in competitive sports. This underlines the importance of futsal research in the global context of sport science and health promotion.

The variation in workload metrics across microcycles has been less studied than the variability observed in competition [5,9,16,17]. Nonetheless, these variations in microcycle workloads are critical for achieving the necessary adaptations in elite team athletes [14].

Poor workload management is a primary injury risk factor in team sports and player availability [8]. To understand variations throughout a competitive season, it is essential to measure and compare workloads during microcycles. A busy schedule of matches and intense training sessions can increase fatigue, thereby elevating the risk of injury [5,14]. Therefore, workload management is vital for tailoring training to each player’s individual needs, ensuring proper recovery and optimal performance on match days [7,18].

In addressing these challenges, sport science continues to evolve, offering practical strategies to improve team performance and mitigate health risks. This study contributes to examining the external loading dynamics of futsal, providing valuable information on the relationship between workload management, performance, and injury prevention.

Consequently, the aim of this study was to compare the external load of each session along competitive microcycles on an elite futsal team, considering the players’ positions and relationship with competition, to provide insights that support optimised performance, injury prevention, and player well-being.

## 2. Materials and Methods

### 2.1. Participants

The external load of 10 elite players (TIER 3) [19] from a First Division team in the Spanish Futsal League was recorded (age 27.5 ± 7 years, height 1.73 ± 0.05 m, weight 70.1 ± 3.8 kg). Goalkeepers were excluded from the study. The average duration of player participation in training sessions was 1:08 ± 00:12 h:min, which included a 15 min warm-up preceding the main part of the session (Table 1).

### 2.2. Study Design

This study used a longitudinal observational design, which was conducted throughout the 2022–2023 season. A total of 30 competitive microcycles were analysed, including 125 training observations and 21 matches (146 in total) for the evaluation and analysis of external loads over a period of 30 weeks. An example of a typical microcycle is described below (Table 2).

The external load of the players was monitored using the OLIVER IMU (Barcelona, Spain) devices (Inertial Measurement Unit), which have been validated in previous studies [20,21]. All players wore the OLIVER device on their calf, with the devices being activated just before the warm-up for training sessions and immediately after the warm-up for matches [5]. The OLIVER signal frequency was set to 27 Hz. To minimise potential device variability, each player was required to consistently use the same device during all training sessions. The data were analysed using the OLIVER software platform (TryOliver Platform 2.42).

All participants were verbally informed about the purpose and procedures of the study, and each provided signed informed consent in accordance with the Declaration of Helsinki. The study was approved by the University’s Research Ethics Committee (CEI-35/2022).

### 2.3. Procedure

Building on previous futsal studies [1,9,13] that analysed the conditional demands of competition, the variables examined in this study included: total distance (m), walking distance 0–6 km/h (m), jogging distance 6.1–12 km/h (m), high-intensity distance 12.1–18 km/h (m), maximum-intensity distance >18.1 km/h (m), high accuracy (m) (>2 m/s^2^), High Dec (m) (>−2 m/s^2^), number of accelerations (>2 m/s^2^), number of decelerations (>2 m/s^2^), and MAX Speed (km/h). Once the training sessions were completed, the data were categorised according to the day of the week relative to the match. MD-5 (5 days before the match), MD-4 (4 days before the match), MD-3 (3 days before the match), MD-2 (2 days before the match), MD-1 (1 day before the match), and MD (match day), based on previously published scientific studies [5,14].

### 2.4. Data Processing

In the pre-processing phase, a Butterworth filter was implemented to remove noise from the signals, selecting the filter parameters specifically to preserve the relevant features of our data while removing unwanted interference.

For certain analyses that require the detection of specific motion patterns, an additional smoothing process was applied using a Hanning window filter, with a window size based on our sampling frequency. No amplification of the signals is performed, as the original data provide adequate levels for our analysis.

Regarding the inherent limitations of IMUs, the accumulation of error (drift) in distance estimation by double integration of acceleration is well known. To address this limitation, pattern detection and machine learning algorithms were employed to segment the signal into discrete intervals, periodically restarting the integration process to mitigate error accumulation (i.e., detection of steps and different player actions, such as ball impact or ball driving) [20].

### 2.5. Statistical Analysis

The homogeneity of variances was initially assessed using Levene’s test. Subsequently, one-way analysis of variance (ANOVA) was performed to identify differences in the dependent variables across the various days of the microcycle and between playing positions. When significant differences were found, Bonferroni post hoc tests were applied to analyse specific changes when variances were homogeneous; otherwise, Dunnett’s T3 post hoc tests were used. The level of statistical significance was set at *p* < 0.05. Finally, the effect size (ES) was calculated to evaluate the statistical significance and to quantify the magnitude of differences between groups, utilizing Cohen’s *d* statistic. The interpretation of Cohen’s *d* was categorized as follows: values >0.2 indicated a small effect, >0.6 a moderate effect, and >1.2 a large effect. All statistical analyses were performed using SPSS software (version 29.0; IBM Corporation, Armonk, NY, USA).

## 3. Results

Table 3 presents the results obtained after comparing the recorded variables in terms of both day of the week and playing position. The competitive load was significantly higher for distances covered at various speeds greater than 6 km/h, and for the maximum speed reached during the match.

Regarding the comparison of loads across the microcycle, MD-1 showed significantly the lowest values for most variables compared with the rest of the week. Additionally, MD-5 was the second session with the lowest intensity, although it showed significantly higher distances covered at lower speeds (0–6 km/h) (*p* < 0.05). Finally, MD-4 and MD-3 were the most demanding training sessions across all variables; for instance, total distance and high accelerations (>2 m/s^2^) were significantly higher in MD-3 than MD-1 (*p* < 0.05, d: 2.37; *p* < 0.05, d: 2.17, respectively) and also both variables were higher in MD-4 than MD-1 (*p* < 0.05, d: 2.08; *p* < 0.05, d: 1.85, respectively), whereas MD-2 demonstrated significant differences with MD-1 for most variables.

Concerning specific positions, pivots accumulated significantly lower loads across most variables, including distances (*p* < 0.05, d: 2.03), accelerations (*p* < 0.05, d: 2.03), and deceleration (*p* < 0.05, d: 1.21), regardless of the session analysed. During competition, they covered less distance at high speeds (12–18 km/h and >18 km/h) (*p* < 0.05 for both, d: 1.51 and 0.98, respectively). Throughout the rest of the microcycle, pivots exhibited significantly lower performance for all variables during MD-5, MD-4, MD-3, and MD-2 (see Table 3). However, for MD-1, significant differences were observed only in accelerations (*p* < 0.05, d: 0.74) and deceleration (*p* < 0.05, d: 1.05).

## 4. Discussion

The aim of this study was to compare the external load of each session along competitive microcycles on an elite futsal team, considering the players’ positions and relationships with competition. A novel aspect of this study was the analysis of the distribution of the absolute training load based on the match day and specific positions across various variables. The main findings were as follows: (a) MD-1 was the day with the lowest load, consistent with its purpose of recovery and tapering, while MD-4 and MD-3 were the days with the highest loads, reflecting their role in physical conditioning and tactical-technical development. (b) Pivots experienced the lowest loads during the microcycle, likely due to their positional role with less total distance covered, whereas wingers and defenders showed the highest loads across the different variables analysed, indicative of their higher physical demands during both defensive and offensive phases of play. These findings underscore the importance of adapting training and recovery strategies not only to optimize performance, but also to reduce the risk of overtraining and injury by addressing critical aspects of an athlete’s well-being and long-term health.

Although research has been conducted in sports such as football [14], volleyball [16], basketball [22], and handball [23], regarding the distribution of training load in relation to match days and specific positions, this approach remains limited in futsal. Previous studies have mainly focused on high-intensity variables [9] or tactical aspects with and without the ball [24], but they have not thoroughly addressed how these variables fluctuate throughout the microcycle based on player positions. This highlights the need for interdisciplinary approaches that bridge the gap between sport science and applied practice and provide practical information to optimize performance and reduce the risk of injury.

The external load data obtained on the matchday were consistent with the values reported in previous futsal studies. The total distance covered in our study (3496.25 ± 1079.96 m) falls within the range reported by earlier research, which varies between 3052 ± 804 m and 3749 ± 1123 m [1,10,11,13]. Similarly, the high-intensity distance (12.1–18 km/h) in this study was 685.44 ± 275.17 m, close to the values recorded during the Final Eight of the Portuguese Cup 2018 (675.3 ± 298.1 m) [1]. Furthermore, the maximum intensity distance (>18 km/h) of 215.18 ± 128.02 m is comparable with previously reported ranges, from 254 ± 101 m [10] to 134.9 ± 54.1 m [1]. These findings demonstrate a consistent profile of external load demands across competitive futsal contexts, underscoring their relevance for broader applications in team management and player conditioning.

These similarities in metrics suggest that despite potential differences in playing style, team structure, and competitive level, the fundamental physical demands in elite futsal matches are fairly consistent. Such consistency provides a solid foundation for evidence-based strategies aimed at improving player performance and promoting long-term health in high-performance environments. It also demonstrates how standardized workload metrics can serve as a critical tool for aligning training and recovery programs with competitive demands. Regarding accelerations (>2 m/s^2^) and decelerations (<−2 m/s^2^), our findings (535.82 ± 197.27 m and 504.15 ± 182.87 m, respectively) also align with values observed in studies conducted on elite teams [13]. Moreover, the number of acceleration (135.93 ± 48.72) and deceleration (134.59 ± 50.17) is almost identical to that reported by teams from the National Futsal League (135 ± 41 and 129 ± 39, respectively) [10]. This finding is particularly significant because it highlights that these high-intensity actions are important to futsal dynamics and should be a focal point in the design of training and recovery programmes. For instance, prioritising exercises that simulate frequent acceleration and deceleration could enhance players’ physical responsiveness during competition, especially during critical moments such as rapid transitions or counterattacks.

These data suggest that competitive loads in futsal exhibit notable consistency among elite Spanish teams and similar leagues, such as the Portuguese league [1]. However, it is essential to note that these metrics could vary depending on contextual factors, such as match duration, tactical approaches, and opposition intensity. Future research that incorporates these contextual elements may provide a more complete and nuanced understanding of external workload patterns, which could lead to advances in team performance management and injury prevention.

Regarding the structuring of the microcycle in our study, MD-1 was the day with the lowest load across all variables (*p* < 0.05). MD-4 was the day with the greatest distance covered, while MD-3 recorded the highest metres covered at high intensity (12.1–18 km/h) and maximum intensity (18.1–3600 km/h). In addition, the day on which the highest meters covered in acceleration and deceleration was MD-4. These results reflect a competitive microcycle profile aligned with common practises in other team sports, where a post-competition recovery session of low to moderate intensity is planned, alongside tapering programming [8]. Such structured approaches highlight the importance of balancing performance optimisation and recovery, reflecting a holistic strategy to manage athletes’ physical and psychological readiness for competition. This strategy aims at progressive reduction of the load from MD-4 to MD-1, thereby optimising recovery and ensuring that athletes arrive in the best possible condition for the next match [25,26,27]. The finding that MD-1 is the day with the lowest load is consistent with previous futsal studies [9], reinforcing the idea of structuring this day as an activation session focused on preparing players for the match day.

When comparing our results with those of other sports, similarities can be found. In handball, the day with the greatest covered distance is typically MD-4, with the highest maximum-intensity runs occurring on the same day [23]. In football, the day with the greatest distance covered during training is MD-3, which differs from our article, which states MD-4 [14,28]. However, our study aligns with soccer research in that the highest distances for maximum-intensity runs were recorded on MD-3 [28]. These inter-sport comparisons not only enhance our understanding of common principles of load management, but also underscore the adaptability of microcycle structuring strategies to the unique demands of each sport. These findings contribute to a multidisciplinary understanding of workload distribution that advances sport science and public health frameworks.

The playing style influences the performance demands of each role, as observed in other sports [14]. In futsal, this research shows that pivots are the players with the lowest loads across different training days and matches, except for MD-1, where the load is similar across all positions. This finding is consistent with those of previous futsal studies [10,29]. This may be attributed to the anthropometric profile of pivots, who tend to have the highest body fat percentage compared with other positions [30].

Wingers are the players who record the highest values in variables such as distance covered, high intensity (12.1–18 km/h), maximum intensity (18.1–3600 km/h), High Acc (m) (>2 m/s^2^), and High Acc count (>2 m/s^2^), results that align with previous research [10]. However, for the variables of High Dec count (>−2 m/s^2^) and High Dec (m) (>−2 m/s^2^), depending on the training day, higher values were observed for both defenders and wingers. In matches, defenders carry the highest load on these variables, although no statistically significant differences were found. These findings emphasise the importance of considering the specific demands of each position when designing training programmes, which has already been proposed in other sports [31].

Nonetheless, in futsal, this study is the first to quantify the magnitude of efforts across the competitive microcycle according to playing position. Future research is therefore needed to confirm whether conditional profiles vary, showing pivots as the players with the lowest workload and wingers and defenders with similar workloads, with minor variations depending on the session. Moreover, based on the findings of this study, it is evident that more personalised and specific training should be developed according to the conditional demands of each position.

In handball, compared with other sports, right-wing players registered the lowest total distance covered and the shortest high-intensity distances during matches, while pivots covered the least distance and performed the fewest high-intensity efforts during training sessions [23]. In football, midfielders and attacking midfielders cover the greatest distances during the microcycle, while central defenders and forwards cover the least. However, the highest maximum-intensity distances during training are recorded by fullbacks, with midfielders covering the least [14]. Lastly, in basketball, pivots recorded the highest number of accelerations, while small forwards executed the greatest number of decelerations [32]. These inter-sport analyses highlight the importance of a multidisciplinary and comparative approach to load management, which not only benefits performance in specific sports but also informs broader sport science and athlete health frameworks. While futsal shares similarities with sports such as handball, football, and basketball, the specific demands of each discipline require further investigation to identify tailored training strategies that maximise the desired adaptations. Such an approach would not only enhance player performance but also reduce the risk of injury, particularly in highly intermittent sports like futsal.

One of the main limitations of this study is the small sample size, as only 10 players from one elite futsal team were included. This may limit the generalizability of the findings to other teams or competitive levels. Additionally, goalkeepers were not considered, thereby excluding a crucial component of the team whose physical and conditional demands are unique. Furthermore, although data from 21 official matches were collected, it was not possible to evaluate the entire 30-match season due to logistical and permission constraints, which may have affected the representativeness of the competitive demands recorded. Another limitation is the lack of contextual analysis considering factors such as match outcome, opponent quality and players’ physical condition, all of which could significantly influence the observed external loads. Finally, the absence of internal load measurements, such as heart rate profiles or ratings of perceived exertion, which would provide a more comprehensive understanding of futsal match-related demands. Future studies should incorporate these parameters to gain deeper insights into the physiological responses during matches [33].

In future research, it would be advisable to expand the sample size to include more teams, competitive levels, and positions such as goalkeepers to enhance the generalizability and relevance of the findings. An extended longitudinal analysis covering multiple seasons could also be valuable, allowing the identification of consistent patterns or variations associated with tactical and physical changes over time. Moreover, incorporating contextual factors such as opponent level, match outcome, and season phase would enrich the understanding of conditional demands. Finally, exploring the relationship between monitored external load patterns during the microcycle and injury risk could help develop more effective prevention strategies.

## 5. Conclusions

The findings of this study highlight that the microcycle structure in an elite futsal team follows a clear pattern, where the day with the lowest external load is MD-1, used as a pre-match activation session, while the days with the highest load are MD-3 and MD-4, depending on the variables analysed. This structured approach reflects the importance of balancing recovery and preparation, ensuring that players are physically, tactically, and mentally optimised for competition. Such strategies align with holistic frameworks in sports science, aimed at sustaining high performance while minimising injury risks. Additionally, the metrics for total distance, intensity, and the number of acceleration and deceleration obtained in this study underline the importance of monitoring these variables to adjust the loads throughout the microcycle.

Regarding positions, the results reveal significant differences in conditional demands. Pivots display the lowest loads during the microcycle, while wingers stand out in variables such as high- and maximum-intensity distance, as well as accelerations, indicating greater physical demands for this position. Defenders exhibit higher loads during decelerations, particularly during matches. These insights emphasize the necessity of designing position-specific training programmes that account for these conditional differences, enhancing player readiness and reducing the risk of overuse injuries.

By offering reference values derived from elite contexts, this study provides practical guidance for S&C coaches, enabling them to plan, adjust, and optimise training programmes in futsal. Moreover, the findings contribute to the broader discourse in sport sciences and public health by addressing critical issues such as workload management, injury prevention, and player well-being. Such multidisciplinary insights are essential for advancing sustainable performance in highly intermittent sports like futsal.

## Figures and Tables

**Table 1:** Duration of each training session along the microcycle.

Session	Duration (h:min) (M ± SD)
MD-5	01:11 ± 00:04
MD-4	01:20 ± 00:09
MD-3	01:16 ± 00:06
MD-2	01:10 ± 00:05
MD-1	00:50 ± 00:02

M: mean. SD: standard deviation.

**Table 2:** Example of a typical competitive microcycle.

Session	Content	Dimension	Time
MD-5 (In the morning)	Gym		
MD-5 (In the afternoon)	Possession	28 × 20	6′
	Warming up goalkeepers	20 × 20	6′
	2 × 1 fragmented track king or every 1′30″ changes	40 × 20	6′
	Set piece + 2 × 2 link + pivot	25 × 20	8′
	Conditioned match	28 × 20	8′
	5 × 4 specific attack	20 × 20	6′
MD-4	Possession	40 × 20	6′
	2 × 2 small space with separate goalkeepers	10 × 10	5′
	3 × 3 3 teams king of the track or every 1′30″ changes	23 × 20	8′
	Conditioned match	40 × 20	8′
	Modulated match	40 × 20	2 × 4′
	5 × 4 specific defense	20 × 20	8′
MD-3	Positional game	20 × 20	5′
	Rondo + regression link	40 × 20	6′
	3 × 2 transitions	40 × 20	4′
	3 × 3 + pivots	25 × 20	6′
	Conditioned match	40 × 20	6′
	Modulated match	40 × 20	7′
MD-2	Positional game	40 × 20	7′
	Warming up goalkeepers	20 × 20	5′
	Conditioned match	40 × 20	2 × 9′
	5 × 4 specific defense	20 × 20	5′
	5 × 4 specific attack	20 × 20	5′
MD-1	Peladao	40 × 20	9′
	Match conditioned for set pieces	20 × 20	9′
	5 × 4 specific attack	20 × 20	9′
MD	Game		
MD+1	Day off		

A conditioned match refers to running time, while a modulated match refers to stopped time.

**Table 3:** Differences in external load between session of microcycle and playing position.

	Position	MD	MD-1	MD-2	MD-3	MD-4	MD-5
		M ± SD	M ± SD	M ± SD	M ± SD	M ± SD	M ± SD
Total distance (m)	Defenders	3434.57 ± 1198.55 b	2705.61 ± 344.38 ^	3787.93 ± 494.15 b,^	4087.06 ± 605.98 a,b,c,f,^	4146.3 ± 731.4 a,b,c,f,^	3587.7 ± 818.92 b,^
	Wingers	3569.08 ± 1169.04 b	2838.71 ± 309.11 °,^	3805.17 ± 635.89 b,^	4169.65 ± 526.92 a,b,c,f,^	4248.5 ± 786.28 a,b,c,f,^	3795.83 ± 662.13 b,^
	Pivots	3439.22 ± 660.25 b,c	2218.33 ± 303.17	3011.88 ± 568.8 b	3320.06 ± 566.25 b	3378.01 ± 690.36 b,c	3124.82 ± 520.57 b
	Total	3496.25 ± 1079.96 b	2659.12 ± 397.99	3615.8 ± 660.97 b	3949.11 ± 657.87 a,b,c,f	4005.41 ± 821.66 a,b,c,f	3555.1 ± 737.51 b
[0–6] km/h (m)	Defenders	1223.7 ± 388.41	1190.24 ± 163.02 ^	1630.95 ± 193.79 a,b,f,^	1591.16 ± 283.31 a,b,^	1648.78 ± 237.87 a,b,f,^	1428.98 ± 330.38 a,b,c
	Wingers	1166.65 ± 336.18	1176.36 ± 174.03 ^	1568.24 ± 201.79 a,b,f,^	1549.15 ± 226.64 a,b,f,^	1599.67 ± 238.9 a,b,f,^	1404.81 ± 239.62 a,b,c
	Pivots	1267.44 ± 282.05 b	1082.83 ± 203.99	1429.59 ± 290.21 b	1429.19 ± 275.63 b	1496.69 ± 252.66 a,b	1380.7 ± 192.12 b
	Total	1208.18 ± 343.18	1161.49 ± 181.21	1558.26 ± 234.59 a,b,f	1536.91 ± 264.87 a,b,f	1592.71 ± 247.59 a,b,f	1407.47 ± 264.96 a,b
[6.1–12] km/h (m)	Defenders	1356.27 ± 525.58 b	1103.08 ± 165.09 ^	1538.71 ± 271.84 b,^	1646.91 ± 274.97 a,b,^	1686.92 ± 352.3 a,b,c,^	1592.66 ± 328.44 b,^
	Wingers	1393.84 ± 490.04 b	1201.32 ± 139.85 °,^	1585.52 ± 270.57 a,b,^	1682.55 ± 230.96 a,b,^	1738.6 ± 368.79 a,b,c,^	1671.14 ± 353.46 a,b,^
	Pivots	1417.55 ± 260.11 b,c,d,e,f	878.67 ± 136.12	1223.5 ± 255.32 b	1345.81 ± 245.75 b	1394.11 ± 357.52 b,c	1338.19 ± 284.08 b
	Total	1387.45 ± 458.4 b	1097.49 ± 191.84	1485 ± 303.65 b	1594.11 ± 283.62 a,b,c	1638.4 ± 384.47 a,b,c	1560.44 ± 352.03 a,b
[12.1–18] km/h (m)	Defenders	676.06 ± 299.52 b,c	383.55 ± 122.48 ^	528.27 ± 145.58 b,^	697.25 ± 183.78 b,c,f,^	685.83 ± 227.73 b,c,f,^	548.93 ± 167.52
	Wingers	757.34 ± 292.57 b,c,f	413.1 ± 93.47 °,^	580.97 ± 153.64 b,°,^	761.03 ± 184.95 b,c,f,^	763.47 ± 248.38 b,c,f,^	620.07 ± 173.98
	Pivots	559.02 ± 117.64 b,c,d,e,f,°,*	228.78 ± 70.51	310.13 ± 109.69 b	449.09 ± 150.96 a,b,c	416.15 ± 184.98 a,b,c	360.20 ± 133.34
	Total	685.44 ± 275.17 b,c,f	363.34 ± 123.27	499.52 ± 177.01 b	668.28 ± 214.54 b,c,f	653.35 ± 264.53 b,c,f	530.13 ± 191.24
[18.1–3600] km/h (m)	Defenders	178.55 ± 92,36 b,c,e,f	28.74 ± 28.14	90 ± 65.45 b,^	151.73 ± 77.6 b,c,f,^	124.77 ± 84.93 a,b,^	90.36 ± 45.37 b,^
	Wingers	251.25 ± 151.39 b,c,d,e,f,°,^	47.93 ± 36.22 °,^	105.35 ± 64.15 b,^	176.91 ± 88.92 a,b,c,f,^	146.76 ± 92.2 a,b,c,f,^	99.82 ± 67.62 a,b,^
	Pivots	195.22 ± 101.21 b,c,d,e,f	28.06 ± 27.71	48.66 ± 45.79 b	95.97 ± 76.55 a,b,c,f	71.06 ± 58.64 a,b	45.74 ± 36.29 b
	Total	215.18 ± 128.02 b,c,d,e,f	36.81 ± 33.1	86.77 ± 64.55 b	149.81 ± 87.69 a,b,c,e,f	120.95 ± 87.49 a,b,c,d,f	83.02 ± 57.51 b
High Acc (m) (>2 m/s^2^)	Defenders	497.6 ± 190.45 b	315.34 ± 67.06 ^	454.71 ± 91.81 b,^	541.38 ± 106.02 b,c,^	568.56 ± 141.62 b,c,^	515.72 ± 130.41 b,^
	Wingers	571.87 ± 220.84 b,c	358.43 ± 60.26 °,^	483.9 ± 124.38 b,^	604.38 ± 113.01 b,c,f,°,^	611.67 ± 150.27 b,c,f,^	545.05 ± 109.38 b,c,^
	Pivots	518.07 ± 141.49 b,c,f	268.26 ± 60.21	354.13 ± 108.49 b	447.64 ± 117.76 b,c	435.46 ± 145.27 b,c	405.67 ± 112.59 b
	Total	535.82 ± 197.27 b,c	323.79 ± 71.5	443.63 ± 120.93 b	546.87 ± 126.5 b,c,f	554.47 ± 161 b,c,f	500.09 ± 129.86 b,c
High Dec (m) (>−2 m/s^2^)	Defenders	511.1 ± 212.51 b	352.4 ± 90.97	487.81 ± 107.73 b,^	562.48 ± 124.86 b,c,^	601.47 ± 143.22 b,c,^	551.36 ± 147.33 b,^
	Wingers	503.01 ± 189.79 b	366.4 ± 90.5	485.11 ± 155.32 b,^	582.17 ± 140.13 a,b,c,^	592.73 ± 162.95 a,b,c,^	563.87 ± 141.33 b,c,^
	Pivots	496.88 ± 116.98 b,c,e	262.06 ± 81.54	346.63 ± 128.57 b	426.74 ± 130.64 b,c	412.12 ± 149.86 a,b	412.56 ± 132.63 b
	Total	504.15 ± 182.87 b,c	339.21 ± 97.41	454.06 ± 145.99 b	540.21 ± 145.97 b,c	552.96 ± 171.57 b,c	522.06 ± 153.97 b,c
High accuracy (>2 m/s^2^)	Defenders	128.92 ± 49.97 b	85.44 ± 16.81 ^	119.6 ± 22.71 b,^	140.43 ± 27.18 b,c,^	148.24 ± 35.37 b,c,^	134.44 ± 33.25 b,^
	Wingers	141.63 ± 53.48 b	94.06 ± 15.21 ^	125.27 ± 31.1 b,^	152.48 ± 28.45 b,c,°,^	155.63 ± 36.94 b,c,f,^	139.69 ± 25.99 b,c,^
	Pivots	134.45 ± 34.92 b,c,f	72.19 ± 15.73	94.92 ± 27.49 b	117.29 ± 27.86 b,c	116.75 ± 36.75 b,c	110.06 ± 28.73 b
	Total	135.93 ± 48.72 b,c	86.32 ± 17.89	116.26 ± 29.95 b	140.31 ± 30.86 b,c	143.77 ± 39.34 b,c,f	130.49 ± 31.6 b,c
High Dec quantity (>−2 m/s^2^)	Defenders	141.08 ± 62.12 b	102.12 ± 26.83 ^	138.86 ± 30.98 b,^	156.24 ± 37.31 b,c,^	168.49 ± 39.39 a,b,c,^	154.12 ± 40.18 b,^
	Wingers	131.41 ± 49.19 b	104.26 ± 27.28 ^	134.99 ± 43.58 b,^	158.54 ± 40.04 a,b,c,^	162.42 ± 44.32 a,b,c,^	156.09 ± 39.75 a,b,c,^
	Pivots	131.94 ± 29.86 b,c	74.27 ± 24.68	98.38 ± 37.84 b	117.44 ± 36.26 b	113.63 ± 41.52 b	116.03 ± 38.85 b
	Total	134.59 ± 50.17 b	97.13 ± 29.05	127.89 ± 41.42 b	148.47 ± 41.63 a,b,c	153 ± 47.29 a,b,c	145.51 ± 42.89 b,c
MAX Speed (km/h)	Defenders	24.62 ± 1.92 b,c,e,f	20.7 ± 2.71	22.75 ± 2.33 b,^	24.09 ± 1.62 b,c,f,^	23.28 ± 2.27 a,b,^	22.83 ± 1.95 b,^
	Wingers	24.64 ± 1.85 b,c,e,f	20.97 ± 2.42	22.6 ± 2.24 b,^	23.8 ± 2.14 b,c,e,f,^	22.6 ± 2.24 a,b,d	22.47 ± 2.27 b,^
	Pivots	25.14 ± 1.71 b,c,d,e,f	20.45 ± 2.73	20.94 ± 2.93	22.2 ± 2.72 a,b	22.03 ± 2.54 a,b	21.26 ± 2.43
	Total	24.75 ± 1.85 b,c,d,e,f	20.76 ± 2.59	22.27 ± 2.55 b	23.54 ± 2.24 a,b,c,e,f	22.71 ± 2.36 a,b,d	22.3 ± 2.27 b

M: mean. SD: standard deviation. a: >MD; b: >MD-1; c: >MD-2; d: >MD-3; e: >MD-4; f: >MD-5; °: >CI, *: >AL, ^: >PV.

## Data Availability

The data from this research can be made available by the corresponding author upon reasonable request. Due to privacy concerns, the data are not accessible to the public.
